# From Chemical Defense to Drug Discovery: Bioactive Molecules, Pharmacological Activities, and Therapeutic Potential of *Rhaebo guttatus* and *Rhinella marina* Secretions

**DOI:** 10.3390/biology15181665

**Published:** 2026-09-20

**Authors:** Beatriz Evilym Dutra Da Silva, Lucinéia Reuse Albiero, Valéria Dornelles Gindri Sinhorin

**Affiliations:** 1 Faculty of Medicine Athens, Sorriso 78890-000, MT, Brazil; beatriz.silva.sorriso@uniatenas.edu.br (B.E.D.D.S.); lucineia.albiero@ufmt.br (L.R.A.); 2 Postgraduate Program in Biotechnology and Biodiversity of the Pró Centro-Oeste Network, Federal University of Mato Grosso, Sinop 78550-728, MT, Brazil

**Keywords:** Bufonidae, *Rhaebo guttatus*, *Rhinella marina*, bufadienolides

## Abstract

Amazonian bufonid toads represent an important source of bioactive compounds with promising pharmacological potential. Among them, *Rhaebo guttatus* and *Rhinella marina* produce complex cutaneous and parotoid gland secretions composed mainly of bufadienolides, indole alkaloids, antimicrobial peptides, biogenic amines, and other secondary metabolites that play essential roles in chemical defense. This narrative review summarizes current knowledge regarding the ecology, chemical composition, biological activities, toxicological profile, and therapeutic potential of secretions from these two Amazonian species. A literature search was conducted using PubMed, Scopus, Web of Science, SciELO, and Google Scholar, covering publications from 2005 to 2025. The reviewed evidence demonstrates that these secretions exhibit a broad spectrum of biological activities, including antiproliferative, antimicrobial, antiparasitic, antiviral, immunomodulatory, antioxidant, anti-inflammatory, analgesic, and larvicidal effects. These pharmacological properties are primarily associated with the modulation of molecular targets such as Na^+^/K^+^-ATPase, intracellular signaling pathways, oxidative stress, and inflammatory mediators. Despite their remarkable therapeutic potential, the clinical application of crude secretions remains limited by their significant systemic toxicity and narrow therapeutic window. Overall, the secretions of *R. guttatus* and *R. marina* constitute a valuable natural reservoir of bioactive molecules with considerable potential for drug discovery and the development of innovative therapeutic agents.

## Simple Summary

Amazonian toads produce complex skin and parotoid gland secretions that serve as an effective defense against predators and microorganisms. These secretions contain a wide variety of bioactive compounds, including bufadienolides, alkaloids, antimicrobial peptides, and other metabolites with diverse biological activities. In recent years, these natural products have attracted increasing scientific interest because of their potential applications in medicine, biotechnology, and agriculture. This narrative review summarizes current knowledge on the chemical composition, biological activities, mechanisms of action, toxicity, and therapeutic potential of the secretions of *Rhaebo guttatus* and *Rhinella marina*. Evidence indicates that these secretions exhibit antiproliferative, antimicrobial, antiparasitic, antiviral, immunomodulatory, anti-inflammatory, antioxidant, and larvicidal properties. However, their therapeutic application remains challenging because several compounds also display significant systemic toxicity. Understanding the relationship between chemical structure and biological activity will be essential for identifying safer and more selective molecules. Overall, the secretions of these Amazonian species represent a valuable source of natural compounds that may contribute to the development of new drugs and other biotechnological products while highlighting the importance of conserving biodiversity as a reservoir of bioactive molecules.

## 1. Introduction

The Amazon region holds one of the greatest biodiversities on the planet, harboring a vast array of anuran amphibians that have developed complex chemical defense mechanisms for survival in their ecological niches [1,2,3]. Among the most representative families, Bufonidae stands out for the presence of specialized cutaneous glands, such as the parotoid glands, capable of synthesizing and storing a secretion rich in biocompounds with distinct pharmacological properties [4,5,6]. Species such as *Rhaebo guttatus* (*R. guttatus*) and *Rhinella marina* (*R. marina*), widely distributed in the Southern Amazon, have attracted increasing scientific interest due to the potential of their secretions as sources of active biocompounds [7,8,9,10,11]. However, although preliminary tests point to antiparasitic activities, the pharmacological use of crude extracts is hampered by a high profile of cellular cytotoxicity [12].

Chemically, the poison of these bufonids is a “cocktail” of secondary metabolites, composed mainly of bufadienolides (cardiotonic steroids) and indole alkaloids [13]. Bufadienolides, such as marinobufagin (MBG) and bufaline, are recognized for their specific interaction with the Na^+^/K^+^-ATPase enzyme, which confers antitumor, cardiotonic, and cell cycle modulating activities [14]. In parallel, the alkaloid fraction, represented by molecules such as bufotenine and dehydrobufotenine, presents a promising profile for the development of analgesic and antiplasmodial agents, offering alternatives for the treatment of neglected diseases such as malaria [15].

Despite the historical recognition of their acute toxicity, which can cause severe poisoning in predators and domestic animals, modern science seeks to elucidate the therapeutic window of these compounds [16]. Recent investigations indicate that these secretions not only inhibit the growth of cancerous cells and pathogens, but also act in modulating the immune response and the antioxidant redox status of the host [16,17]. However, the complexity of these interactions and the inherent toxicity of crude extracts necessitate detailed studies of the molecular mechanisms involved.

In this context, the present work proposes an in-depth review of the biological and pharmacological activities of the secretions of *R. guttatus* and *R. marina*. The analysis ranges from antiproliferative and antimicrobial potential to systemic effects of toxicity and oxidative modulation, aiming to consolidate current knowledge and highlight the strategic value of Amazonian fauna for drug bioprospecting and the advancement of biotechnology.

## 2. Methodology

This study consists of a narrative literature review aimed at compiling and discussing current knowledge regarding the ecology, chemical composition, and biological activities of the cutaneous and parotoid gland secretions of *Rhinella marina* and *Rhaebo guttatus*, with emphasis on their pharmacological and biotechnological potential.

The literature search was conducted between January and May 2026 using the electronic databases PubMed, Scopus, Web of Science, SciELO, and Google Scholar, published between the years 2005 and 2025. The search strategy employed combinations of the following keywords and their respective Boolean operators: “*Rhinella marina*”, “*Rhaebo guttatus*”, “Bufonidae”, “parotoid secretion”, “cutaneous secretion”, “bufadienolides”, “alkaloids”, “chemical composition”, “antimicrobial activity”, “antiparasitic activity”, “antitumor activity”, “immunomodulatory activity”, “pharmacological potential”, and “Amazonian amphibians”.

Original research articles, review papers, book chapters, and relevant reports published in English, Portuguese, or Spanish were considered. Preference was given to peer-reviewed publications addressing the chemical characterization of secretions, ecological aspects of the species, and experimentally validated biological activities of compounds isolated from Bufonidae amphibians.

Studies not directly related to *R. marina*, *R. guttatus*, or closely related species of the family Bufonidae were excluded when their findings could not contribute to the understanding of the ecological, biochemical, or pharmacological aspects discussed in this review. Classical studies of historical relevance and recent publications were included to provide both a foundational and updated overview of the subject.

The selected literature was analyzed qualitatively and organized into thematic sections covering species ecology and distribution, glandular morphology, chemical composition of secretions, ecological functions of bioactive compounds, and their reported pharmacological applications. Given the narrative nature of this review, no formal systematic review protocol or meta-analysis was performed.

## 3. Taxonomic Aspects, Geographic Distribution and Ecology

The genus *Rhaebo*, described by Edward Drinker Cope in 1862, belongs to the family Bufonidae, which currently comprises about 471 species distributed across 33 genera [18]. Currently, *Rhaebo* consists of nine species of neotropical frogs, distributed from eastern Honduras to the South American Amazon, encompassing countries such as Colombia, Ecuador, Peru, Bolivia, Venezuela, the Guianas, and Brazil [2]. Recent records indicate the presence of these species in Amazon–Cerrado transition areas, such as in the southern Brazilian Amazon, reinforcing the importance of these regions as sympatric zones with other bufonids, especially *R. marina* [19].

**Figure 1:**
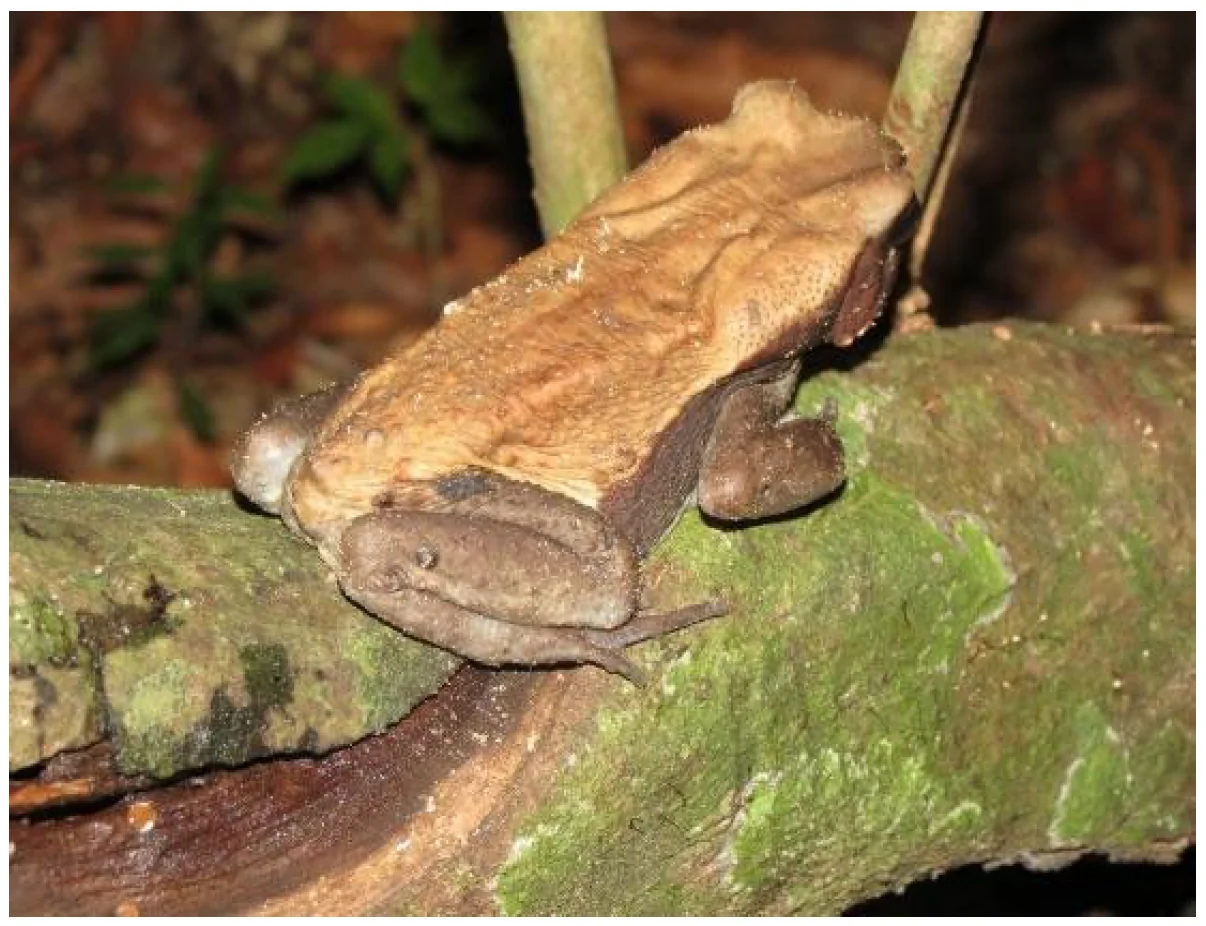
*Rhaebo guttatus*, Xingu Reserve, Mato Grosso, Brazil. Photograph by Marcos Penhacek, kindly provided for use in this study. Photograph taken on 22 September 2022.

The genus exhibits diagnostic morphological characteristics that allow differentiation between its species: *R. guttatus* (Figure 1) has a well-developed preocular crest, a brown to dark brown back, a belly with rounded cream spots and light groins, and oval and prominent parotoid glands. In contrast, *R. glaberrimus* has smoother skin, lacks a preocular crest, and has inconspicuous parotoid glands. Other species, such as *R. ecuadorensis*, show distinct combinations of skin color and texture, reinforcing the morphological diversity of the genus [2].

In addition to morphological characteristics, acoustic signals have proven important for taxonomic differentiation. In *R. guttatus*, the release call, recently described in the Brazilian Amazon, features short, simple harmonic notes, distinct from the advertisement call, suggesting behavioral adaptations to specific environments and providing information about the species’ reproductive ecology [20].

From an ecological point of view, species of the genus *Rhaebo* preferentially occupy humid tropical forests and non-flooded upland areas, often associated with leaf litter near small watercourses [2]. The cutaneous secretion, produced mainly by the parotoid glands, acts in defense against predators and pathogenic microorganisms. In some species, such as *R. guttatus*, the chemical composition of this secretion can be influenced by diet and environment, especially with regard to alkaloids, while steroids appear to be more conserved [3,21].

**Figure 2:**
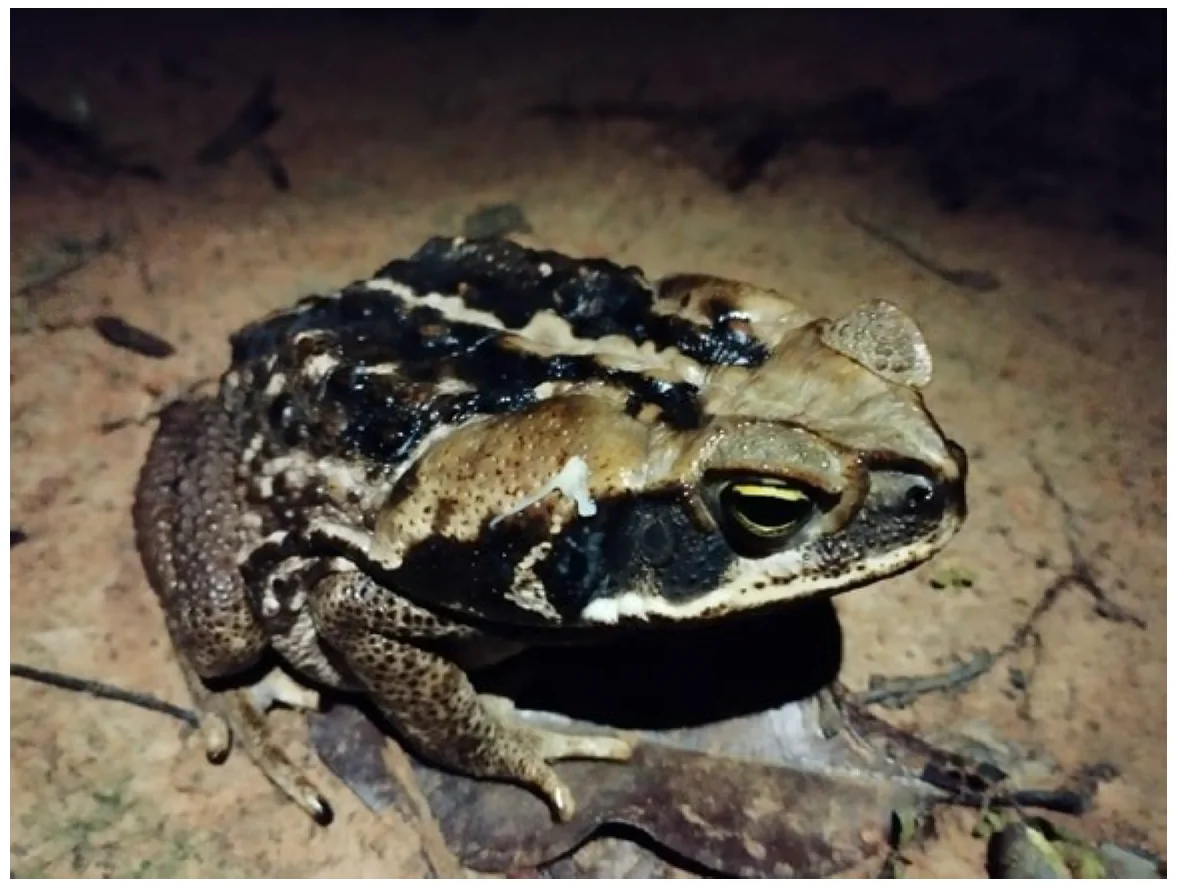
*Rhinella marina*, Xingu Reserve, Mato Grosso, Brazil. Photograph by Marcos Penhacek, kindly provided for use in this study. Photograph taken on 22 September 2022.

The genus *Rhinella*, formerly included in Bufo, is the most representative of the Bufonidae family, with over 250 recognized species [4,11]. It stands out for its great morphological and ecological plasticity, adapting to a wide diversity of habitats, from humid forests to open and anthropized areas. Emblematic species, such as *R. marina* (Figure 2*)*, can exceed 20 cm in snout-vent length and 1 kg in body mass, presenting rough and warty skin of olive-brown to dark brown coloration, a whitish to yellowish belly, voluminous and elongated parotoid glands, and well-developed cephalic crests from the preorbital to the occipital region [2,4]. Species of the *Rhinella diptycha* group demonstrate morphological variation related to different phytophysiognomies, such as the Pantanal, Chaco, and Cerrado, indicating that the geographic diversification of the genus was influenced by the formation of the large South American hydrographic basins during the Tertiary and Quaternary periods [22].

The distribution of *Rhinella* extends from southern Texas and Mexico, throughout Central and South America, to the Brazilian Amazon [19]. *R. marina* exhibits high ecological plasticity, occurring in Amazonian floodplain environments, open areas, and anthropized zones, while *R. diptycha* is observed aggregating in river ports in the Pantanal, a behavior associated with the abundance of insects attracted by artificial light [23,24,25]. These species show a preference for bodies of water for reproduction and daytime refuge in humid environments, but are capable of exploring altered habitats [26].

In the southern Brazilian Amazon, simultaneous records of *R. guttatus* and *R. marina* confirm the occurrence of sympatry between the genera in transition zones, reinforcing the importance of this region for studies of ecology, distribution, and conservation of bufonids [19]. The diversity of habitats occupied and the morphological and behavioral variability of these genera highlight their ecological and evolutionary relevance, evidencing specific adaptations to micro-habitats and historical processes of diversification in tropical America.

## 4. Chemical Composition of Cutaneous and Parotoid Secretions

The secretions produced by the exocrine glands distributed throughout the skin of frogs and toads, and particularly conspicuously by the parotoid macroglands characteristic of the Bufonidae family—including the neotropical genera *Rhaebo* and *Rhinella*—represent a chemical arsenal of high complexity and functional diversity [5,6,13]. These mixtures, rich in secondary metabolites, constitute a sophisticated chemical defense mechanism, essential for the survival and ecological success of these amphibians, providing protection against a wide spectrum of biological threats, from vertebrate and invertebrate predators to a myriad of potentially pathogenic microorganisms (bacteria, fungi, viruses, protozoa) prevalent in their diverse habitat [5,27,28]. Detailed analysis of these secretions has revealed a rich molecular tapestry, highlighting major classes of bioactive compounds, including: cardiotonic steroids known as bufadienolides (exemplified by MBG, telocinobufagin, and helveticoneolin); various alkaloids, with emphasis on indolic and tryptamine derivatives (such as bufotenine and dehydrobufotenine); a variety of bioactive peptides and proteins with antimicrobial and immunomodulatory properties; as well as biogenic amines, such as serotonin and catecholamines [10,13,14,15].

The synthesis and storage of these defensive substances occur primarily in the granular (or poison) glands of the dermis, which in Bufonidae frequently aggregate to form externally visible macroglands. The parotoid glands, located bilaterally in the postorbital region, are the most developed and characteristic [5,6,8,10]. These structures function as strategic reservoirs, capable of rapidly releasing their contents—viscous secretions, typically whitish or yellowish—in response to direct mechanical stimuli, such as the pressure exerted by a predator’s bite, or in situations perceived as threatening [5]. The exact release mechanism, however, exhibits remarkable interspecific variation reflecting different defensive strategies. In species such as *R. marina*, defense is predominantly passive: external pressure applied to the parotoid gland is necessary to rupture the epithelial plugs that seal the glandular ducts, resulting in the expulsion of poison and self-poisoning of the aggressor. In striking contrast, *R. guttatus* has developed a unique form of active defense among known toads, being able to voluntarily eject jets of poison over considerable distances (up to 2 m). This peculiar mechanism is facilitated by specific morphological adaptations, including the adhesion of the ventral portion of the parotoid gland to the scapula, the absence of a calcified dermal layer over the parotoid gland (conferring greater flexibility), and the presence of structurally weaker ductal plugs, which require less internal pressure (possibly generated by muscle contraction associated with the shoulder girdle and lung inflation) to rupture and release the poison [5].

Chemically, biosynthesis reflects the origin of the precursors: Lipophilic metabolites, notably bufadienolides and other cardiotonic steroids, derive from cholesterol metabolism. Nitrogenous components, such as tryptamines (bufotenin, serotonin) and other indole alkaloids, originate from the tryptophan biosynthetic pathway, while peptides (such as bufonins, dermaseptins, defensins, and the potent buforin II from *Bufo gargarizans*) are products of gene expression [13]. In addition to these main classes, free (non-steroid-conjugated) diacyl arginine derivatives, such as suberoyl and azelayl arginine, and other biogenic amines such as histamine have been identified in *Rhinella* [29]. The final chemical composition is not only species-specific, reflecting phylogeny, but also demonstrates intraspecific plasticity, being modulated by ecological (habitat, diet—especially for sequestered alkaloids), physiological (reproductive stage, age, sex), and environmental (temperature, humidity) factors, resulting in locally adapted chemical profiles [3]. The reproductive physiology of *R. guttatus* has also been experimentally investigated, including hormonal induction and cryopreservation approaches for reproductive material, highlighting the relevance of physiological state when interpreting biological variation in this species [30]. This chemical and functional complexity underscores the evolutionary and pharmacological potential of these secretions. Functionally, different classes of compounds contribute to distinct biological effects: bufadienolides can exert cardiotoxic effects primarily through inhibition of Na^+^/K^+^-ATPase, whereas alkaloids, peptides, and amines have been associated with neuroactive and antimicrobial activities, respectively [13,14]. This chemical and functional complexity underscores the evolutionary and pharmacological potential of these secretions.

### 4.1. Bufadienolides and Cardiotonic Steroids

Bufadienolides represent the predominant and most studied chemical class in the cutaneous and parotoid secretions of frogs belonging to the genera *Rhaebo* and *Rhinella*, and are also found in certain plant families, such as the Crassulaceae [13]. Structurally, they are defined as C-24 steroids, biosynthesized from cholesterol, characterized by the presence of an α-pyrone ring (a six-membered α, β-unsaturated lactone) fused to the C-17β position of the cyclopentanoperhydrophenanthrene steroid nucleus, which is frequently polyhydroxylated [13,14]. In nature, they can occur as free aglycones or as glycosides, conjugated to sugar residues (commonly L-rhamnose or D-glucose), typically at the C-3 position [14]. Its most notable biological activity is cardiotonic, mediated by the specific inhibition of the Na^+^/K^+^-ATPase enzyme in the cell membrane, a mechanism shared with plant cardenolides such as digitoxin [14,19].

Inhibition of Na^+^/K^+^-ATPase by bufadienolides results in an increase in intracellular sodium concentration ([Na^+^]i), which secondarily leads to an accumulation of intracellular calcium ([Ca^2+^]i) via the Na^+^/Ca^2+^ exchanger [13,19]. In cardiomyocytes, this increase in [Ca^2+^]i elevates the force of contraction (positive inotropic effect), but at excessive or prolonged concentrations, it causes arrhythmias and severe cardiotoxicity, this being the main toxic effect observed in toad poisoning. The biosynthesis of these steroids occurs primarily in the parotoid glands, following complex metabolic pathways from cholesterol, possibly regulated by factors such as environmental stress and the availability of lipid precursors [14].

Detailed chromatographic (HPLC-UV, LC-MS/MS) and spectrometric analyses allowed the identification of several bufadienolides in the species of interest. In *R. marina*, a consistently more complex profile is observed, with the marked presence of telocinobufagin, marinobufagin, bufalin, and resibufogenin as main components, in addition to conjugates such as marinobufoxin (3-(N-suberoylargininyl) marinobufagin) [10]. MBG frequently stands out as the major constituent, representing up to 60% of some extracts. In contrast, *R. guttatus* exhibits a simpler chemical profile in this class, with MBG being the predominant bufadienolide or, in some analyses, the only one detected in significant quantities, although other minor steroids such as 3β,16β-dihydroxybufa-8(14),20,22-trienolide and bufatrienolides have also been identified [3,14].

In addition to their potent cardiotonic activity, bufadienolides demonstrate a broad spectrum of other bioactivities that support their pharmacological interest. Of particular note is their strong cytotoxic and antiproliferative activity against various human tumor cell lines in vitro (leukemia, breast, lung, colon, ovary, glioblastoma, melanoma, liver), often superior to or comparable to chemotherapeutic agents such as doxorubicin [8,14,19]. The antitumor mechanism often involves the induction of apoptosis, associated with mitochondrial depolarization, generation of reactive oxygen species (ROS), DNA fragmentation, and cell cycle arrest [13,14]. Other important pharmacological activities include anti-inflammatory effects [31], antiviral effects (against HBV, HIV, coronavirus), antibacterial effects, antifungal effects (including against phytopathogens and *Batrachochytrium dendrobatidis*), antiparasitic effects (against Leishmania, Trypanosoma, Plasmodium), immunomodulatory effects, and, in some cases, insecticidal and larvicidal effects (as observed against *Anopheles darlingi*) [12,13,15,31].

Ecologically, bufadienolids are crucial for chemical defense. Their intensely bitter taste and toxic effects act as a powerful deterrent against predation by vertebrates [5,13]. Contact with secretions can cause severe irritation of the mucous membranes, salivation, vomiting, cardiac arrhythmias, and, in smaller or more sensitive predators, even death, promoting rapid aversive learning [13,14].

### 4.2. Tryptamine Alkaloids and Derivatives

Alkaloids and their tryptamine derivatives constitute a relevant chemical fraction, although generally less abundant than bufadienolides, in the cutaneous and parotoid secretions of frogs of the genera *Rhaebo* and *Rhinella*. These nitrogenous compounds, biosynthesized mainly from the amino acid tryptophan through specific enzymes, are characterized by their high neurophysiological and pharmacological activity [13,14]. Their actions include neurotoxic and psychotropic (hallucinogenic) effects, and modulation of neurotransmission systems, particularly serotonergic receptors, due to their structural similarity to serotonin. During chromatographic analysis, these compounds tend to concentrate in the more polar (hydrophilic) fractions of the extracts [14].

Among the alkaloids most frequently identified in these genera, bufotenine (5-hydroxy-N, N-dimethyltryptamine) and its dehydrogenated derivative, dehydrobufotenine, stand out, both consistently reported in several species, including *R. marina*, *R. diptycha*, and *R. guttatus* [14]. Bufotenidine, a quaternary ammonium derivative of bufotenine, was also found in *R. diptycha* [28]. Other tryptamines include N-methyl-5-hydroxytryptamine (identified in *R. guttatus*) and serotonin itself (5-hydroxytryptamine), detected in both main species (*R. marina* and *R. guttatus*). Additionally, the potent psychedelic tryptamine 5-methoxy-N, N-dimethyltryptamine (5-MeO-DMT) and bufotionine have been mentioned as constituents of bufonid secretions [13]. Although generally present as minor components in terms of mass (<5% of total metabolites in some analyses), their high biological potency gives them significant functional and pharmacological importance [15].

The biological activities associated with these alkaloids are varied and noteworthy. Bufotenine, despite its known hallucinogenic properties that limit its direct therapeutic application, has demonstrated in vitro the ability to inhibit rabies virus infection, possibly through interaction with nicotinic acetylcholine receptors. Studies also suggest that it possesses anti-inflammatory and analgesic properties [13]. Dehydrobufotenine has exhibited antiproliferative activity against some cell lines [15], antifungal activity (including against phytopathogens such as *Alternaria solani* and *Botrytis cinerea*), and has emerged as a promising prototype for the development of antiplasmodial drugs, demonstrating activity against *Plasmodium falciparum* with a favorable profile of low cytotoxicity and high selectivity. Synthetic analogs of dehydrobufotenine have shown even greater potential as antifungal and antiviral agents against tobacco mosaic virus. Serotonin, in turn, is a known biogenic amine with neuromodulatory and vasoconstrictive functions [12,15,26].

In an ecological context, the presence of these alkaloids, particularly those with psychotropic and neurotoxic effects, plays a crucial role in chemical defense against predators [14]. Contact with secretions rich in these compounds can induce rapid and intense aversive responses in the aggressor, such as excessive salivation, disorientation, vomiting, and convulsions, facilitating the prey’s escape and promoting strong avoidance learning on the part of the predator [13,14]. This mechanism contributes to the adaptive success of species such as *R. marina*, which efficiently colonizes diverse environments, including anthropized areas. Evidence also suggests that the production or modification of some alkaloids may be related to the symbiotic skin microbiota of the frog or dietary sequestration of precursors [13]. Despite pharmacological interest, especially in antimicrobial, antiviral, and antiparasitic activities, the systemic toxicity and psychotropic effects of tryptamines such as bufotenine represent significant challenges for clinical development, reinforcing the need for more structural, pharmacokinetic, and structure–activity relationship studies to explore the therapeutic potential of these nitrogenous metabolites from neotropical anurans [13,15].

### 4.3. Antimicrobial Peptides and Other Metabolic Classes

In addition to bufadienolides and alkaloids, which frequently dominate the chemical characterization of Bufonidae secretions, a diverse set of bioactive peptides and other classes of secondary metabolites contributes significantly to the complexity and functionality of these defensive mixtures [14]. Peptides, in particular, represent a crucial component of the innate cutaneous immunity of anurans, forming an essential chemical barrier against colonization and infection by opportunistic and pathogenic microorganisms, aiding in the maintenance of microbial homeostasis of the skin [27]. Although less explored in *Rhaebo* and *Rhinella* compared to other anuran families, the granular glands of these frogs also synthesize and secrete peptides, generally of low molecular weight (1–5 kDa). These include members of families known as dermaseptins, bufonins, temporins, defensins, and ranatensins, many of which exhibit broad-spectrum antimicrobial activity against Gram-positive and Gram-negative bacteria, as well as fungi [27].

Experimental studies confirm the functional relevance of these peptides in Bufonidae secretions. Methanolic extracts of *R. guttatus* secretion have demonstrated significant antifungal (against phytopathogens such as *Fusarium solani*, *Aspergillus flavus*) and antibacterial (*Escherichia coli*, *Staphylococcus aureus*) activity in vitro [27]. Inactivation assays using heat or protease treatment confirmed that this antimicrobial activity is substantially associated with the peptide fraction of the secretion. In *R. marina*, cationic peptides with structural characteristics similar to magainins and bombinins were detected, suggesting a mechanism of action based on the destabilization and lysis of the microbial cell membrane [27]. A notable example of a potent AMP isolated from a bufonid (*Bufo gargarizans*) is buforin II, a histone H2A-derived peptide that exhibits exceptional broad-spectrum antimicrobial activity through an unusual mechanism of membrane translocation and subsequent binding to intracellular nucleic acids. Cathelicidins have also been identified in the same species [27].

In addition to defense against microorganisms, many peptides derived from frogs also exhibit selective cytotoxicity against tumor cells [19]. This effect is frequently mediated by the induction of apoptosis, either through preferential interaction with cancerous cell membranes or by mitochondrial membrane depolarization. The antiproliferative activity observed in crude extracts of *R. guttatus* against breast and liver cancer cell lines [8] can be partially attributed to these peptides, which possibly act synergistically with the bufadienolides and alkaloids coexisting in the secretion [14]. This co-occurrence of multiple classes of toxins with distinct mechanisms of action suggests an integrated and robust defensive strategy, where peptides can potentiate or complement the action of steroids and alkaloids, amplifying the overall effectiveness of secretion and hindering the development of resistance by predators or pathogens [27].

Other minor, yet functionally relevant, metabolic classes include various biogenic amines, in addition to the tryptamines already discussed. Catecholamines such as adrenaline and dopamine are also known constituents of bufonid secretions. An additional intriguing class, identified in *R. marina* and *R. diptycha*, is diacyl arginine derivatives that exist in free form (not esterified to bufadienolides), such as adipoyl, pimeloyl, suberoyl, azelayl, and sebacyl arginine esters. Argininyl bufadienolide esters have also been detected in *R. diptycha* [29]; the specific function of these compounds in secretion still needs elucidation. Proteins, detected by electrophoresis, make up another significant fraction and may play structural, enzymatic, or homeostatic roles [14]. Lipids, such as glycerides and fatty acids, can influence the physicochemical properties of secretion, while phenolic compounds could contribute to antioxidant properties [4].

Taken together, the concomitant presence of antimicrobial and cytotoxic peptides, cardiotonic steroids, neuroactive alkaloids, biogenic amines, and other metabolites gives the secretions of *Rhaebo* and *Rhinella* an exceptionally integrated and dynamic chemical profile [14,27]. Each metabolic class plays potentially synergistic and complementary roles in the organism’s defense against a wide range of biological threats. This complex chemical arrangement is the result of long evolutionary processes shaped by multiple selective pressures—predation, microbial competition, abiotic stresses—that favored the diversification and optimization of adaptive chemistry in these neotropical anurans [13,14]. Beyond their undeniable ecological importance, the ongoing elucidation of the structure and function of peptides and other minor metabolic classes in these secretions represents a promising frontier for the discovery of new antimicrobial, antitumor, immunomodulatory, and antioxidant agents with potential biotechnological and pharmaceutical applications [4,14].

### 4.4. Intra- and Interspecific Chemical Variations

The chemical composition of the defensive glandular secretions of frogs belonging to the genera *Rhaebo* and *Rhinella* exhibits remarkable heterogeneity, manifesting itself both between distinct species (interspecific variation) and within the same species (intraspecific variation). The latter can occur between geographically isolated populations, between individuals of the same population, between sexes, or even throughout the ontogenetic development of an individual. This chemical variability is the complex result of the interaction between genetic inheritance (reflecting evolutionary history and gene flow), ecological factors (such as habitat type, availability and composition of diet—crucial for sequestered metabolites, the local community of predators and pathogens), abiotic environmental conditions (climate, temperature, humidity) and the physiological state of the animal (age, size, body condition, reproductive cycle) [13,14]. The study of these variations is fundamental not only for the chemical taxonomy and understanding of the evolutionary ecology of these amphibians, but also guides more efficient bioprospecting strategies for the discovery of new bioactive molecules [14].

At the interspecific level, differences in chemical composition can be striking and serve as auxiliary diagnostic characters in species delimitation, especially in morphologically complex or cryptic groups. Although the formal description of species such as *Rhaebo ecuadorensis* has been based primarily on morphological characters that distinguish it from *R. guttatus* (e.g., absence of a preocular crest) and *R. glaberrimus* (e.g., body size, groin coloration) [2], it is highly likely that these morphological distinctions are accompanied by specific chemical profiles. A comparison between the well-studied chemical profiles of *Rhinella marina* and *Rhaebo guttatus* reveals substantial and consistent differences [14,17]. *R. marina* typically exhibits a more complex cocktail of bufadienolides (including telocinobufagin, bufalin, and resibufogenin, in addition to MBG and a greater diversity of conjugated derivatives, such as diacid arginine esters [13,29].

In contrast, *R. guttatus* demonstrates a simpler bufadienolide profile, dominated by MBG, with other steroids present in smaller or variable amounts [17]. This fundamental chemical divergence may be directly correlated with the distinct defensive strategies observed (predominantly passive in *R. marina* vs. active ejection capability in *R. guttatus*) [5] and with the differences in the biological effects of their poisons (greater systemic lethality associated with *R. marina* vs. greater induction of pain and local edema by *R. guttatus*). Distinct alkaloid profiles also contribute to interspecific differentiation [14]. Intraspecific variation, within the same species, is equally significant and offers insights into adaptive plasticity and the environmental influence on defensive chemistry [14]. A key comparative study involving *R. guttatus* populations from different regions of the Brazilian Amazon (preserved primary forest vs. Amazon–Cerrado transition zone with greater anthropogenic influence) compellingly illustrated this point [17]. While the chromatographic profile of steroids (bufadienolides) showed remarkable consistency between individuals from both locations, indicating a strong endogenous basis and genetic control for their biosynthesis, the alkaloid composition exhibited marked variability [17]. Individuals from the preserved forest showed a more uniform pattern, while those from the transition zone showed highly variable and non-standardized alkaloid profiles. This dichotomy strongly suggests that the accumulation and/or modification of alkaloids in *R. guttatus* is significantly influenced by external factors, such as diets (probable sequestration of precursors from consumed arthropods, a mechanism known in Bufonidae and other anuran families) and local environmental conditions. In *R. marina*, intraspecific variations have also been documented, correlating different bufadienolide profiles with geographic variations (humid vs. dry habitats), seasonality (possible fluctuations associated with the reproductive cycle), and perhaps even with the degree of anthropization of the environment [14]. Factors such as age, body size, and sex also modulate composition; for example, it has been observed in *R. guttatus* that females tend to have higher concentrations of steroids, while males may present higher levels of alkaloids and peptides, suggesting differential roles linked to reproductive physiology or sex-specific defensive strategies.

In conclusion, the chemical heterogeneity observed in the glandular secretions of *Rhaebo* and *Rhinella*, both between and within species, is a multifactorial phenomenon that reflects the complex interaction between evolutionary inheritance, physiological plasticity, and ecological pressures [14]. Detailed analysis of these variations provides valuable information on the taxonomy, chemical ecology, and adaptive strategies of these anurans. Furthermore, the structural diversity resulting from this variability represents a natural resource of immense value for pharmacology and biotechnology, offering a vast field for the discovery of new molecules with therapeutic potential in diverse areas, from cardiovascular diseases and cancer [8,14] to the development of new antimicrobial [27], antiparasitic [12], and larvicidal agents [31].

## 5. Biological and Pharmacological Activities

The cutaneous and parotoid secretions of frogs from the Bufonidae family, particularly the species *R. guttatus* and *R. marina*, represent one of the most complex and promising chemical arsenals of the Amazonian fauna [4,17]. Historically recognized for their acute toxicity, these secretions have been the subject of intense bioprospecting investigations, revealing a wide range of bioactivities that transcend the primary function of defense against predators [13]. The pharmacological versatility of these poisons is due to the presence of distinct secondary metabolites, mainly bufadienolides (cardiotonic steroids) and indole alkaloids, which selectively interact with cellular receptors and metabolic pathways in mammals [15,27]. A summary of the main bioactive compounds identified in *R. guttatus* and *R. marina*, together with their reported biological activities, experimental models, and proposed mechanisms of action, is presented in Table 1.

The recent literature highlights that the biological efficacy of these extracts does not stem solely from a single component, but from a synergistic action between their nitrogenous and steroidal fractions [14]. While bufadienolides are widely recognized for their potent cytotoxic and cardiotonic activity via inhibition of the Na^+^/K^+^-ATPase enzyme, alkaloids such as bufotenine and dehydrobufotenine demonstrate relevant analgesic and antiparasitic properties [13,15]. Additionally, in vivo studies have revealed the ability of these poisons to modulate the host’s immune system and redox status, alternating between protective and oxidative stress-inducing effects, depending on the dosage and tissue context [16].

### 5.1. Cytotoxic and Antiproliferative Activity

The cytotoxic potential against neoplastic cells is the most prominent and documented pharmacological property of bufonid secretions, signaling a promising path for the development of new cancer treatments [8,19]. This antitumor effect is directly correlated with the high concentration of bufadienolides [13], whose antiproliferative efficacy has already been demonstrated in vitro by crude extracts of *R. marina* and *R. guttatus* against breast cancer cell lines (MDA-MB435) and liver cancer cell lines (HepG2), with a dose-dependent pattern [8].

The underlying mechanism of action operates at multiple molecular levels. The primary and most studied target is the Na^+^/K^+^-ATPase enzyme. Bufadienolides bind to this membrane ion transporter, which is frequently overexpressed in tumor cells, transforming it from a transporter into a signaling receptor. This binding triggers a signal transduction cascade involving the activation of Src tyrosine kinase (proto-oncogene tyrosine-protein kinase Src) and MEK kinase (mitogen-activated protein kinase kinase), culminating in the activation of the ERK1/2 pathway (extracellular signal-regulated kinase 1/2). This critical axis is responsible for disrupting ionic balance, proliferation, and cell survival. MBG, a bufadienolide researched in this context, exemplifies the depth of this action by inducing specific cellular changes in human neoplasms [14]. Biochemically, MBG causes DNA cleavage into oligo-nucleosomal fragments, a signature of programmed cell death, and demonstrates potent cytotoxicity against cell lines such as promyelocytic leukemia (HL-60) and colon carcinoma (HCT-116). Morphologically, treated cells exhibit cellular and nuclear shrinkage, chromatin condensation, and apoptotic body formation, as well as cytoskeletal disorganization. At the cell cycle level, MBG prevents orderly cell division, altering the progression of the G1/S/G2/M phases.

Bufadienolides have demonstrated antiproliferative and cytotoxic effects against several cancer cell lines. In particular, newly identified bufadienolides from *R. marina* inhibited Na^+^/K^+^-ATPase and induced apoptosis in human breast and ovarian cancer cells, accompanied by activation of caspases-3 and -9 [32,33]. In addition to inhibiting Na^+^/K^+^-ATPase, bufadienolides induce cell death through well-defined internal pathways. Bufaline, for example, demonstrates pathway specificity: In human leukemia cells U937, its apoptotic action is dependent on the activation of the AP-1 protein [19], a transcription factor involved in cell proliferation and death. In contrast, in human osteosarcoma MG-63 cells, bufaline acts directly on the intrinsic (mitochondrial) pathway, promoting an imbalance between pro- and anti-apoptotic proteins through the downregulation of Bcl-2/Bax [14], which triggers the release of cytochrome C and the subsequent activation of the caspase cascade. Although the molecular mechanisms have not yet been fully elucidated, these compounds have been associated with the inhibition of cell proliferation and the induction of programmed cell death in various tumor cell lines, reinforcing the pharmacological potential of anuran secretions as a source of bioactive molecules for the development of new antineoplastic agents [19].

In summary, the cytotoxicity of the secretion is the result of a complex and synergistic action [14]. Although bufadienolides (including conjugated derivatives such as argininyl esters) in *R. diptycha* [29] are the main culprits, bioactive peptides also contribute significantly, exhibiting selective cytotoxicity through interaction with carcinogenic membranes [19]. Additionally, extracts of *R. marina* and *R. guttatus* exhibit antimutagenic activity, suggesting potential protection against mutagen-induced genetic damage [19].

### 5.2. Immunomodulatory and Anti-Inflammatory Activity

**Figure 3:**
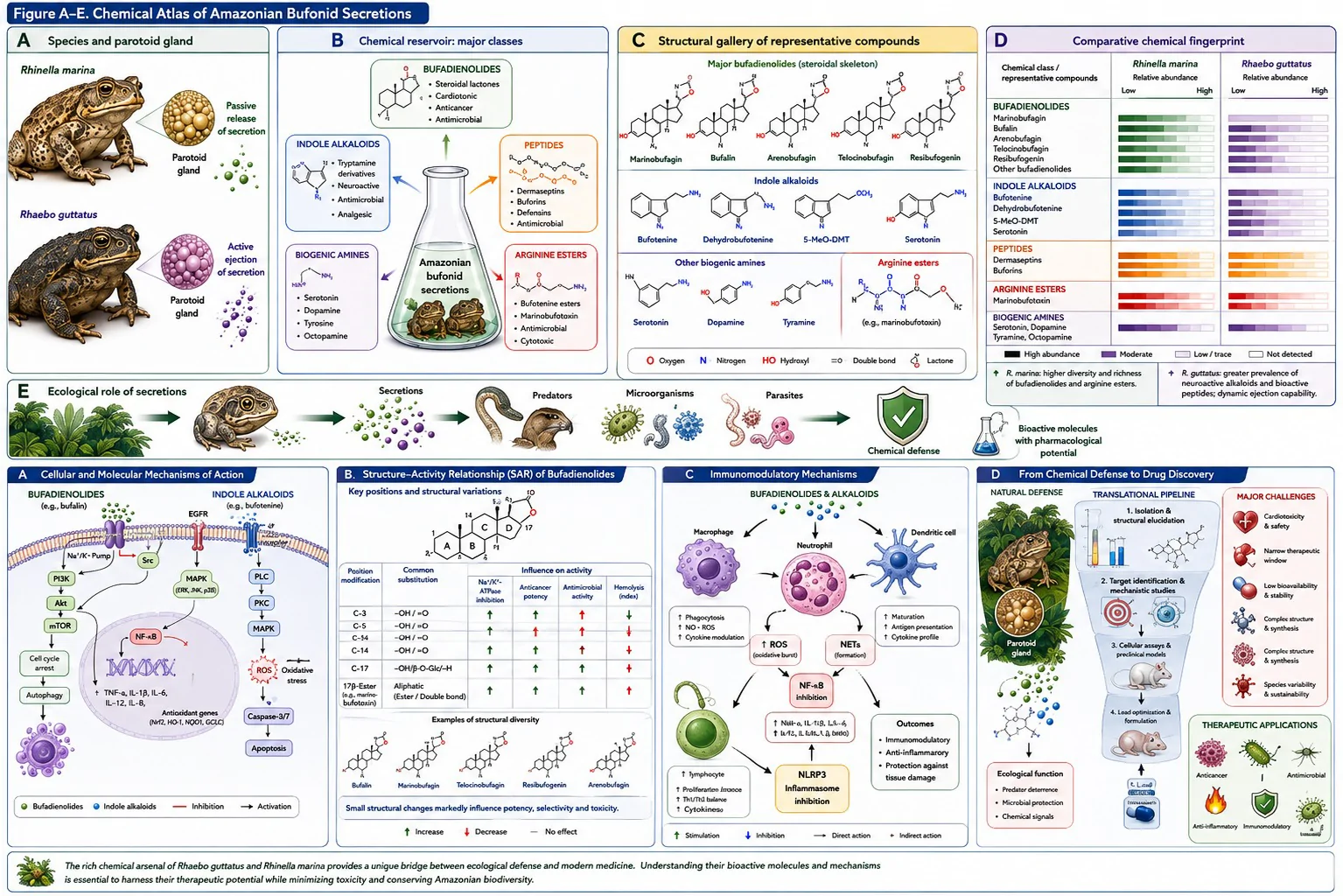
Proposed molecular mechanisms underlying the immunomodulatory effects of *Rhinella marina* and *Rhaebo guttatus* venoms. The illustration was generated with the assistance of artificial intelligence using ChatGPT (OpenAI), based on the concepts and mechanisms described in this review.

The glandular secretions of *Rhaebo* and *Rhinella* act as potent modulators of the immune response, exhibiting a functional duality that varies between stimulating host defense mechanisms and suppressing exacerbated inflammatory processes. This immunomodulation is mediated by the complex interaction between bufadienolides, alkaloids, and peptides with cells of the immune system, especially macrophages and lymphocytes [4,13]. Recent evidence further supports this immunomodulatory potential, demonstrating that venom extracts from *Rhinella marina* and *Rhaebo guttatus* modulate responses in human peripheral blood monocytes [34]. The glandular secretions of *Rhaebo* and *Rhinella* act as potent modulators of the immune response, exhibiting a functional duality that varies between stimulating host defense mechanisms and suppressing exacerbated inflammatory processes. This immunomodulation is mediated by the complex interaction between bufadienolides, alkaloids, and peptides with cells of the immune system, especially macrophages and lymphocytes (Figure 3).

Studies conducted with the crude methanolic extract of *R. guttatus* secretion revealed a significant capacity to stimulate innate immunity. In vitro, the extract was able to activate peritoneal macrophages, resulting in a dose-dependent increase in the production of reactive oxygen species (ROS) and the release of crucial pro-inflammatory cytokines, such as Tumor Necrosis Factor alpha (TNF-α) and Interleukin-12 (IL-12) [35]. IL-12 plays a fundamental role in bridging the gap between innate and adaptive immunity, being essential for the differentiation of helper T cells into the Th1 phenotype, which enhances the antitumor response and the fight against intracellular pathogens [31]. This stimulatory effect appears to be mediated by the interaction of cardiotonic steroids, such as telocinobufagin and bufaline, which promote the polarization of macrophages towards the M1 phenotype (pro-inflammatory/antitumor), activating signaling pathways involving the Na^+^/K^+^-ATPase/Src complex [35].

Conversely, the secretions also exhibit remarkable anti-inflammatory and analgesic properties, often associated with their nitrogenous fraction. Indole alkaloids such as bufotenine, identified in both *R. marina* and *R. guttatus*, demonstrate anti-inflammatory action through the inhibition of lipid metabolism pathways involved in the synthesis of inflammatory mediators [13]. Additionally, some bufadienolides have the ability to suppress the secretion of pro-inflammatory cytokines in specific cell lines, possibly through modulation of the NF-κB signaling pathway, suggesting therapeutic potential for the treatment of chronic inflammatory diseases [4].

The immunomodulation exerted by these secretions is not limited to cell activation, but also extends to the regulation of cell adhesion. It has been found that signaling via Na^+^/K^+^-ATPase induced by these cardiotonic steroids can increase the expression of adhesion molecules on endothelial and immune cells, facilitating the recruitment of leukocytes to sites of inflammation or antigenic challenge [35]. However, the clinical application of these properties requires caution, since excessive stimulation of inflammatory cytokines can lead to tissue damage, highlighting the need to isolate specific compounds that maintain immunomodulatory efficacy with reduced systemic toxicity [4,35].

### 5.3. Antioxidant Activity and Modulation of Oxidative Stress

The cutaneous secretion of bufonid exhibits a complex interaction with the host’s redox system, acting both as a direct antioxidant agent and as a potent modulator of systemic enzymatic defenses [4]. Recent studies conducted with the crude methanolic extract of *R. guttatus* in in vivo experimental models (2024) revealed that exposure to these substances significantly alters the oxidative status, with pronounced impacts on liver tissue [16].

Biochemically, the poison of *R. guttatus* promotes the modulation of critical parameters of the first line of defense against free radicals. In the liver, an adaptive response was observed, mainly directed at the glutathione system, evidenced by a significant alteration in the activity of Glutathione S-Transferase (GST), signaling an effort by the organ to promote the detoxification of secondary metabolites and xenobiotics. In contrast, Catalase (CAT) activity did not show significant variations compared to the control, suggesting a limitation or consumption of this enzymatic pathway in the initial phase of exposure. Furthermore, when the initial endogenous protective capacity is exceeded, cellular damage occurs due to oxidation. This phenomenon was evidenced in liver tissue by a transient increase in TBARS (thiobarbituric acid reactive substances) levels at 7 days, which confirms the occurrence of lipid peroxidation—the degradation of cell membranes caused by the attack of free radicals [13,16].

The intrinsic scavenging activity demonstrated by extracts of *R. marina* and *R. guttatus* is mainly attributed to the presence of phenolic compounds and indolic alkaloids, such as bufotenine [4,13]. On the other hand, the pro-oxidant effect observed at high doses is related to the action of bufadienolides. By inhibiting the Na^+^/K^+^-ATPase pump, these molecules unbalance the mitochondrial ion flow, exacerbating the production of Reactive Oxygen Species (ROS). This mechanism is, paradoxically, one of the cornerstones of its antitumor efficacy, since the induced oxidative stress leads to DNA fragmentation and programmed cell death of neoplastic cells [4,14].

In summary, the redox activity of this secretion is characterized by a biological duality that depends on the experimental model and the exposure time: while in vitro assays indicate that bufadienolides act as potent inducers of oxidative stress to promote the death of neoplastic cells, systemic in vivo evaluation reveals a complex hepatic modulation. In this organ, exposure causes acute and transient oxidative stress in the initial phase (7 days) characterized by a reduction in reduced glutathione (GSH) and induction of lipid damage, which is mitigated at 30 days by an adaptive response of the antioxidant defense system, evidenced by a compensatory increase in GST activity [4,13,16].

### 5.4. Antimicrobial, Antiparasitic, and Antiviral Activity

The skin secretions of bufonid amphibians constitute a primary line of chemical defense against microorganisms and parasites, presenting a vast array of molecules with therapeutic potential for neglected and infectious diseases [13]. The antimicrobial efficacy of these secretions derives from the synergistic action between antimicrobial peptides (AMPs) and cardiotonic steroids, which compromise the integrity of biological membranes and interfere with essential metabolic pathways [27].

In the field of parasitology, one of the most significant documented advances involves activity against *Plasmodium falciparum*, the etiological agent of malaria. Investigations with toad poisons from the Southern Amazon revealed that both the crude extract and isolated molecules possess potent anti-plasmodial action. Dehydrobufotenine, an alkaloid isolated from *R. marina*, has proven to be a promising prototype molecule, exhibiting selective cytotoxicity against the parasite without severely compromising host cells [15]. The suggested mechanism involves interaction with the membrane of the infected erythrocyte and inhibition of the progression of the parasite’s asexual stages [12,15]. Furthermore, bufadienolides isolated from other *Rhinella* species have already demonstrated in vitro efficacy against *Leishmania* sp. and *Trypanosoma cruzi*, reinforcing the broad-spectrum antiparasitic potential of this family [3,29].

Activity against disease vectors is also a highlight in the recent literature. Extracts of *R. guttatus* and *R. marina* exhibited significant larvicidal activity against the mosquito *Anopheles darlingi*, the main vector of malaria in Brazil [33]. This effect is particularly relevant for controlling mosquito populations in endemic areas, offering a biological alternative to synthetic insecticides [31].

Regarding antimicrobial activity, methanolic extracts of *R. guttatus* and *R. marina* have demonstrated the ability to inhibit the growth of phytopathogens of agricultural interest, such as fungi and bacteria that affect crops of great economic importance [30]. In the human clinical context, bufadienolides exhibit bacteriostatic and bactericidal properties against Gram-positive and Gram-negative strains, in addition to displaying antiviral potential. Reviewed studies indicate that these molecules can interfere with the replication of viruses, such as *Herpes simplex* and even certain influenza subtypes, although the exact mechanisms of viral entry and inhibition are still under detailed investigation [13,27].

It is concluded that the chemical fraction intended for the antimicrobial and antiparasitic defense of bufonids is a rich source of lead compounds. However, the transition from in vitro studies to clinical applications depends on modulating the inherent toxicity of bufadienolides and further understanding the mechanisms of biological selectivity [15,27].

### 5.5. Other Activities (Neuroactivity, Cardiovascular, Analgesics and Others)

In addition to their antitumor and immunomodulatory properties, the secretions of *Rhaebo* and *Rhinella* possess a wide range of effects on vital physiological systems, particularly on the cardiovascular and central nervous systems. These activities are mainly mediated by the presence of cardiotonic steroids and indolic alkaloids, whose chemical structures allow specific interactions with receptors and ion channels in mammals [13].

Concerning neuroactivity and analgesia, the alkaloid fraction plays the main role. Bufotenine and dehydrobufotenine, isolated from *R. marina*, exhibit a structural similarity to serotonin (5-HT), allowing them to bind to serotonergic receptors in the central nervous system [15]. Recent studies indicate that bufotenin possesses significant analgesic and anti-inflammatory properties, acting through the inhibition of lipid metabolism pathways involved in nociception. Additionally, in animal models, these substances can induce sedation or behavioral changes, depending on the dose and route of administration, raising possibilities for the development of new neurological agents. Other biological activities of interest include potential wound healing and hemostatic properties; certain bufadienolides and peptides present in these secretions can accelerate tissue regeneration and modulate blood coagulation, properties that have been historically explored in traditional Asian medicine (such as in the use of ChanSu) and are now beginning to be validated by modern pharmacological assays [13].

In summary, the chemical diversity of the secretions of *R. guttatus* and *R. marina* allows these poisons to act as multi-target modulators. Although the risk of systemic toxicity is high, the isolation and structural modification of molecules such as dehydrobufotenin and MBG represent a promising frontier for the synthesis of drugs with specific cardiovascular and neurological action [13,14,15].

## 6. Toxicity, Adverse Effects and Toxic Mechanisms of Action

The toxicity of the secretions of *R. guttatus* and *R. marina* is a direct consequence of their biological defense function, determined by the presence of cardiotonic steroids and neuroactive alkaloids that can cause severe and sometimes fatal poisoning in mammals [5]. The clinical picture of poisoning is characterized by a rapid progression of symptoms affecting the gastrointestinal, cardiovascular, and neurological systems [13].

The primary adverse effects result from the systemic inhibition of Na^+^/K^+^-ATPase by bufadienolides. In the cardiovascular system, an excess of these molecules causes serious arrhythmias, bradycardia, atrioventricular block, and ventricular fibrillation, mimicking a digitalis overdose [8,14]. In the gastrointestinal tract, irritation of the mucous membranes from direct contact with the poison causes sialorrhea (excessive salivation), vomiting, and intense abdominal pain. In the nervous system, the presence of alkaloids such as bufotenine can induce disorientation, convulsions, mydriasis, and, in cases of high exposure, respiratory arrest [13].

Recent in vivo studies have detailed specific organic oxidative modulation. Research with mice exposed to *R. guttatus* extract demonstrated that, although the organism activates long-term adaptive antioxidant responses, the initial exposure generates a scenario of oxidative stress in liver tissue. The increase in biomarkers such as TBARS at 7 days indicates that acute toxicity manifests through lipid peroxidation and degradation of cell membranes in the liver, accompanied by the temporary consumption of non-enzymatic defenses [16].

In addition to systemic liver toxicity, genotoxicity is a concern. Although the extracts exhibit antimutagenic properties under certain conditions, direct exposure of healthy cells to high concentrations of MBG causes DNA fragmentation and cytoskeletal disorganization, making the use of the crude extract unfeasible without rigorous isolation and purification processes [14].

Therefore, the therapeutic window for the use of compounds derived from these secretions is narrow. Pharmacological safety depends on the isolation and rational modification of specific molecules—such as dehydrobufotenin for antiparasitic purposes—and the development of bufadienolide derivatives with improved selectivity and reduced cardiotoxicity [13,15,36,37].

**Table 1:** Main bioactive compounds identified in the cutaneous and parotoid gland secretions of *Rhinella marina* and *Rhaebo guttatus*, their biological activities, experimental models, and corresponding references.

Species (Toad)	Compound Class	Elucidated/Studied Molecules	Biological Activity	Experimental Model	Main Mechanism	Reference
*Rhaebo guttatus*	Bufadienolides and steroid derivatives	Marinobufagin-related compounds, bufadienolides identified by LC-MS/HPLC	Cytotoxic, antiproliferative, antioxidant effects	Cancer cell lines; HPLC chemical profiling	Steroid glycosides interfere with cellular proliferation and survival pathways	[3,8,10,17,19,35,38]
	Peptides and proteins	Bioactive peptides identified by proteomic/peptidomic analysis	Potential antimicrobial and pharmacological activity	Proteomic LC-MS/MS characterization	Peptide-mediated interaction with microbial/cellular targets	[17]
	Bufadienolides	Bufadienolide profile	Quantitative chemical characterization	HPLC-UV	Identification of steroid toxin composition	[10]
	Crude methanolic extract	Steroids and secondary metabolites	Antioxidant response modulation	Mouse model	Regulation of antioxidant enzymes and redox balance	[16]
	Crude methanolic extract	Steroid compounds and unidentified metabolites	Pro-inflammatory immunomodulation	Mouse peritoneal macrophages	Increased reactive species production and cytokine release	[35]
*Rhaebo guttatus* and *Rhinella marina*	Crude venom extracts	Complex mixture of bufadienolides and metabolites	Antiproliferative activity	Human tumor cell lines	Cytotoxicity and inhibition of cell proliferation	[8]
	Skin secretion metabolites	Unspecified secondary metabolites	Antimutagenic and cytotoxic effects	Mutagenicity assays and cell models	Reduction in DNA damage and cytotoxic effects depending on concentration	[19]
	Venom extracts	Chemical constituents	Antiplasmodial and cytotoxic effects	*Plasmodium* assays; cancer cell models	Parasite inhibition and cellular toxicity	[12]
*Rhinella marina*	Bufadienolides (cardiotonic steroids)	Marinobufagin, marinobufagenin, telocinobufagin, bufalin, cinobufagin, resibufogenin	Cytotoxic, antiproliferative, immunomodulatory, cardiotonic	Human cancer cell lines; enzymatic assays; murine models	Na^+^/K^+^-ATPase inhibition; activation/inhibition of PI3K/Akt, MAPK pathways; induction of apoptosis	[4,9,10,14,23,32,33,34,35,36,37,38,39,40]
	Bufadienolides	Marinobufagin	Antitumor activity	Human cancer cells (neoplastic models) and plant cells	Cell cycle arrest, morphological alterations, oxidative stress induction	[14]
	Bufadienolides	Bufalin, marinobufagin and related bufadienolides	Anticancer activity	Molecular/cellular cancer models	Apoptosis induction, inhibition of tumor growth pathways	[9,36,37]
	Bufadienolides	Marinobufagenin	Cytotoxic and antiproliferative activity	Human tumor cell lines	Modulation of Na^+^/K^+^-ATPase signaling and intracellular pathways	[14,23,39,40]
	Indole alkaloids	Dehydrobufotenin	Antiplasmodial activity	*Plasmodium falciparum* model	Interference with parasite survival pathways	[15]
	Bufadienolides	Argyninyl bufadienolide esters	Antiproliferative activity	Human cancer cell lines	Induction of apoptosis and inhibition of cell proliferation	[29,38]
	Peptides	Novel antimicrobial peptides from skin secretion	Antimicrobial activity	Microbial assays	Membrane permeabilization and disruption of microbial integrity	[40]
	Proteins, enzymes and low molecular weight compounds	Proteomic profile of parotoid secretion	Antimicrobial, enzymatic and biological effects	Biochemical assays	Multiple pathways involving enzymatic and toxic components	[32]
	Steroids, alkaloids, peptides	Chemical profile of skin secretion	Cardiovascular effects	Murine heart model	Modulation of cardiac contractility; interaction with ion transport mechanisms	[18]
	Bufadienolides	Marinobufagin, bufalin and related compounds	Cardiotoxicity	Murine cardiovascular models	Na^+^/K^+^-ATPase inhibition leading to altered Ca^2+^ handling	[18,39]
	Bufadienolides	Major toxins mapped in parotoid glands	Chemical distribution and toxin localization	DESI-MSI imaging	Spatial distribution of toxic metabolites in glands	[41]
	Bufadienolides	Bufadienolide profile	Quantitative chemical characterization	HPLC-UV analysis	Identification and quantification of toxic steroid compounds	[10]
	Crude methanolic extract	Bufadienolides, phenolics and other secondary metabolites	Antioxidant and immunomodulatory activity	Chemical assays; immune cell assays	Modulation of oxidative stress and inflammatory responses	[4]
	Bufadienolide derivatives	Synthetic modified bufadienolides	Reduced cardiotoxic anticancer candidates	Molecular pharmacology models	Structural modification reducing cardiac toxicity while maintaining antitumor activity	[37]

## 7. Conclusions

This review highlights the promising multi-target biological potential of the secretions of *R. guttatus* and *R. marina*, particularly the antiproliferative, antiparasitic, and agricultural activities associated with their bufadienolides and alkaloids. However, translation into therapeutic applications remains limited by the systemic toxicity, limited selectivity, and narrow therapeutic windows reported for several crude extracts and isolated compounds. Future research should therefore prioritize the isolation and structural characterization of individual bioactive molecules, followed by systematic pharmacokinetic and pharmacodynamic investigations, including absorption, distribution, metabolism, excretion, tissue exposure, and target engagement. In parallel, structure–activity relationship (SAR) studies and rational chemical modification should be pursued to identify structural determinants of efficacy and toxicity and to generate derivatives with improved selectivity and therapeutic indices. Comparative in vitro and in vivo toxicity studies should also be integrated early in the drug-development pipeline, with particular emphasis on cardiotoxicity and off-target effects associated with bufadienolides. For antiparasitic and agricultural applications, future studies should prioritize standardized extracts or purified molecules, dose–response relationships, selectivity toward target organisms, environmental safety, and efficacy in relevant in vivo or field models. Finally, standardized extraction procedures, chemical fingerprinting, reproducible bioassays, and pharmacological validation using well-characterized molecular targets will be essential to ensure reproducibility and facilitate translational development. Collectively, these research priorities may enable the rational optimization of amphibian-derived molecules and support the development of safer and more selective pharmacological or biotechnological applications.

## Data Availability

No new data were created or analyzed in this study. Data sharing is not applicable.

## Abbreviations

AMP	Antimicrobial peptide
ATPase	adenosine triphosphatase
Bcl-2/Bax	*B-cell lymphoma 2*/BCL-2-associated protein X
CAT	Catalase
DNA	Deoxyribonucleic acid
ERK1/2	Extracellular Signal-Regulated Kinase 1 and 2
G1/S/G2/M	Gap 1, synthesis, and Gap 2 of interphase, and mitosis of the cell cycle
GSH	Reduced glutathione
GST	Glutathione S-Transferase
HBV	Hepatitis B virus
HCT-116	Human colorectal carcinoma cell line
HIV	Human immunodeficiency virus
HL-60	Human promyelocytic leukemia cell line
HPLC-UV	High-Performance Liquid Chromatography with Ultraviolet Detection
IL-12	Interleukin-12
LC-MS/MS	Liquid Chromatography–Tandem Mass Spectrometry
M1	Pro-inflammatory macrophages
MBG	Marinobufagenina
MDA-MB435	Human melanoma cell line
MEK	Mitogen-activated protein kinase kinase
MG-63	Human osteosarcoma cell line
NF-κB	Nuclear factor kappa B
PI3K/Akt/mTOR	Phosphoinositide 3-kinase/Protein Kinase B/Mechanistic Target of Rapamycin
ROS	Reactive Oxygen Species
SAR	Structure–activity relationships
TBARS	Thiobarbituric acid reactive substances
Th1	T helper 1 cells
TNF-α	Tumor necrosis factor-alpha
